# Supplementary material for: Aromatypicity of Austrian Pinot Blanc Wines
Source: Molecules. 2020 Dec 3;25(23):5705. doi: 10.3390/molecules25235705 (PMC7729673; doi:10.3390/molecules25235705)
Supplement: Supplementary file 1 [file molecules-25-05705-s001.pdf]

Aromatypicity of Austrian Pinot blanc wines

Supplementary Materials:

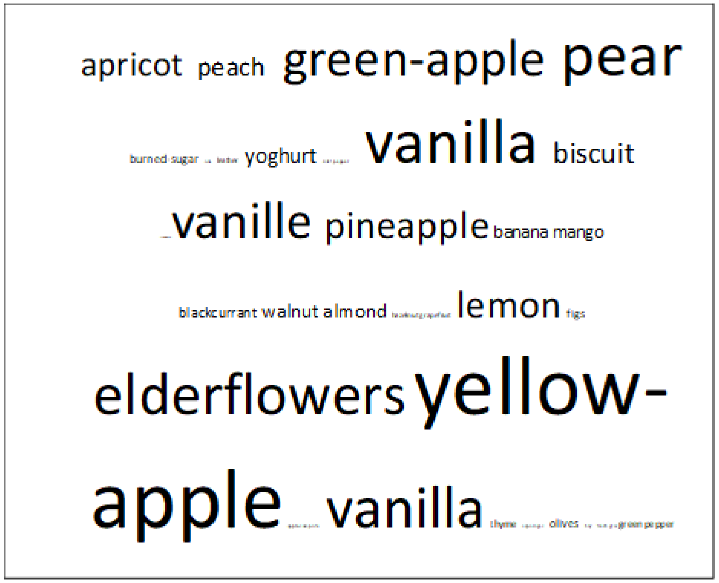

Figure S1. Word cloud of typical attributes for Austrian Weißburgunder, generated by a survey of consumers (N=198).

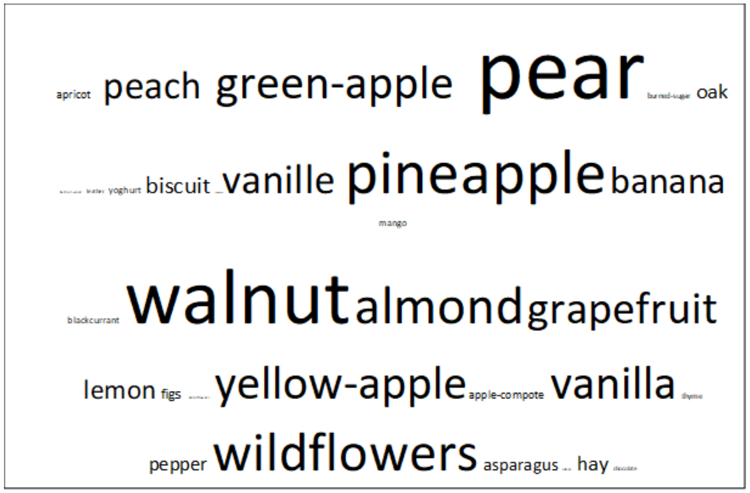

Figure S2. Word cloud of typical attributes for Austrian Pinot Blanc, generated by a survey of producers and experts (N=85).

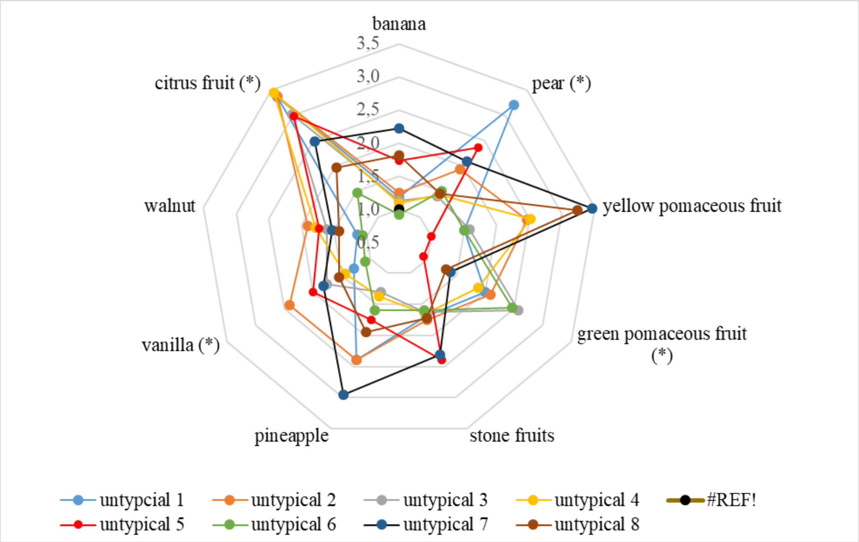

Figure S3. Tasting results of eight atypical Pinot Blanc samples: *indicates a significant difference based on a Kruskal Wallis test ($\alpha \leq 0.05$).

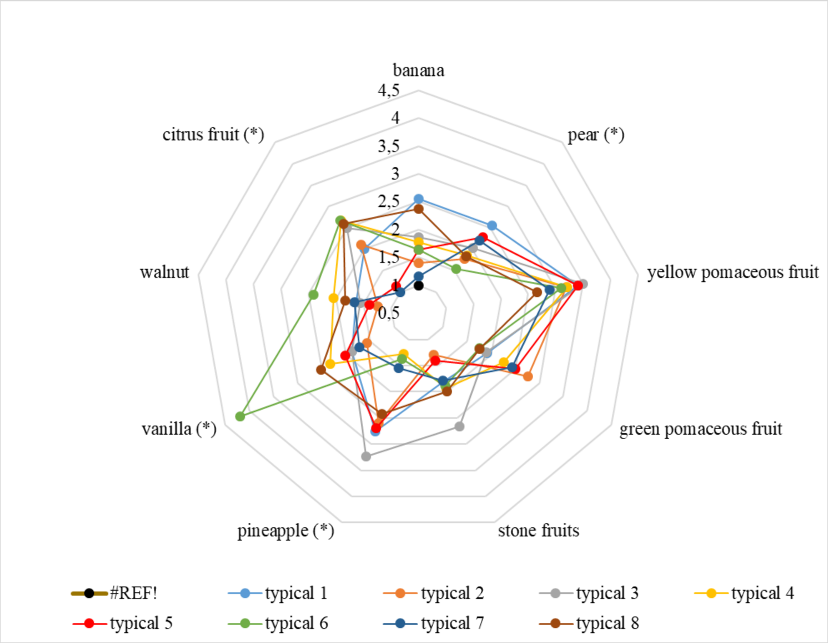

Figure S4. Tasting results of eight typical Pinot Blanc sample: *indicates a significant difference between samples based on a Kruskal Wallis test ($\alpha \leq 0.05$).

Table S1. Concentrations of volatile compounds: Mean values of vintages and origins as well as significant effects (vintage, origin, vintage x origin), significant differences are marked with different letters.

Volatile substances	Average content for vintage			Average content for origin (generic wine region)				Signiciance level		
Free monoterpenes (μg/l)	2015	2016	2017	Lower Austria	Burgenland	Styria	Vienna	vintage	origin	vin * ori
(E)-linalool oxide	2.7	2.6	2.3	2.3	2.2	3.0	3.0			
Linalool	18.2b	8.9a	10.3a	10.6	8.4	18.2	14.7	**		*
Hotrienol	14.5	11.9	9.3	9.8	8.9	18.3	14.8			
nerol oxide	1.3	1.1	0.9	0.9	1.0	1.7	1.3			
alpha-terpineol	9.0a	13.5b	8.4a	8.4	11.2	14.6	12.6	**		*
Nerol	2.4a	6.0b	1.9a	3.6	2.9	4.3	3.1	***		
Citronellol	2.1a	4.6b	4.0b	3.7	3.5	4.3	3.7	***		
Geraniol	17.1b	12.9a	28.2c	20.1	18.1	20.8	19.8	***		**
C6 compounds (μg/l)										
(Z)-3-hexen-1ol	0.08a	0.09a	0.12b	0.10ab	0.08a	0.12b	0.08a	***	*	
1-hexanol	0.77a	1.65c	1.09b	1.28	1.12	1.21	1.07	***		
Higher alcohols (mg/l)										
1-propanol	33.83	33.49	32.78	34.95	30.94	30.66	33.72			
Isobutanol	36.35	34.84	36.65	33.08a	34.81a	36.09ab	48.85b		*	
isoamyl alcohol	153.24	148.33	154.15	153.88	147.88	143.45	156.33			
1-butanol	0.16	0.19	0.18	0.19ab	0.21b	0.13a	0.19ab		*	
Volatile fatty acids (mg/l)										
butyric acid	1.01a	1.35c	1.74b	1.44	1.46	1.37	1.28	***		
isovaleric acid	0.45	0.43	0.41	0.42	0.45	0.37	0.47			
hexanoic acid	2.07b	1.94b	1.34a	1.80	1.52	1.66	2.00	**		
octanoic acid	5.04a	4.71a	5.81b	5.24	5.46	4.92	5.39	**		
isobutyric acid	1.14	1.26	1.12	1.11	1.12	1.24	1.45			
decanoic acid	2.04	2.02	1.90	2.03	1.83	2.01	1.93			
Ethyl esters (μg/l)										
Ethyl acetate (mg/l)*1	48.88b	46.02b	31.25a	44.27	43.44	36.15	30.47	**		
Ethyl propanoate	124.5a	116.0a	250.6b	166.0	202.3	161.8	152.0	***		
Ethyl butanoate	449.5	367.5	398.2	417.4	376.5	379.6	395.6			
Ethyl valerate	0.7a	0.7a	0.9b	0.8ab	0.8ab	0.6a	0.9b	***	*	

Ethyl isovalerate	13.1a	13.8a	17.6b	13.8	18.2	15.7	15.4			*
Ethyl hexanoate	830.8	827.2	733.2	828.9	763.1	716.5	777.2			
Ethyl heptanoate	1.0b	0.6a	0.5a	0.6a	0.7a	0.5a	1.1b	***	*	**
Ethyl octanoate	2804.4b	2118.9a	1933.6a	2349.4	2096.0	2158.0	2058.6			
Ethyl decanoate	1178.8a	1257.4a	1580.4b	1444.8	1230.5	1328.3	1250.3	***		
Ethyl dodecanoate	49.1a	55.0ab	64.9b	61.7	49.8	54.1	53.4	*		
Ethyl tetradecanoate	15.2	11.0	12.8	13.2	11.2	12.7	13.3			
Ethyl hexadecanoate	37.6	32.2	37.4	36.3	32.6	36.4	36.2			
Ethyl lactate (mg/l)	18.65	39.49	22.22	22.98a	27.23ab	50.53b	19.02a	*	**	
Methyl esters (μg/l)										
Methyl hexanoate	1.4b	0.9a	1.5b	1.3	1.3	1.1	1.2	***		
Methyl octanoate	0.9	1.0	0.9	0.9	0.9	0.8	0.9			
Methyl decanoate	0.6a	1.1c	0.8b	0.9	0.8	0.8	0.8	***		
Methyl vanillate	8.8a	11.0ab	12.4b	11.3	10.2	9.6	12.0	***		
Isoamyl esters (μg/l)										
Isoamyl acetate*1	1655.3	1580.0	1849.0	1945.5	1448.5	1409.8	1463.2			
Isoamyl butanoate	0.5	0.6	0.5	0.5	0.5	0.4	0.7			
Isoamyl hexanoate	2.4	1.8	1.6	2.0	1.7	1.7	2.0			
Isoamyl octanoate	2.2a	3.6b	5.4c	4.1	3.8	3.8	3.7	***		
Aromatic esters (μg/l)										
Ethyl benzoate	0.2	0.2	0.3	0.3ab	0.2ab	0.3b	0.2a		*	
Ethyl phenylacetate	2.3	2.3	1.8	1.9a	1.9a	3.4b	1.5a		*	
Higher alcohol acetates (μg/l)										
2-Methylbutyl acetate	110.1	126.4	116.4	132.2	98.9	87.7	123.6	**		
Hexyl acetate	130.0	154.3	136.9	160.2	121.7	118.5	121.7			
Isobutyl acetate	111.5b	110.4b	67.3a	91.2ab	72.4a	91.7ab	132.2b	***	***	
Mixed and other esters (μg/l))										
Isobutyl propionate	17.2a	24.4b	15.9a	18.8	20.9	17.9	20.0	***		***
Butyl isobutanoate	0.2a	0.4b	0.4b	0.4	0.3	0.3	0.4	*		
Pentyl butanoate	0.2	0.2	0.2	0.2ab	0.2ab	0.2a	0.3b		**	
Hexyl isobutanoate*1	0.2a	0.3b	0.3b	0.3ab	0.3ab	0.3a	0.4b	*	*	
Butyl butanoate	13.9b	12.1a	12.2a	12.5	12.6	13.2	12.5	***		
Propyl octanoate	0.9	1.1	1.1	1.1	0.8	0.9	1.1	*		
Isobutyl octanoate	0.6	0.7	0.7	0.7a	0.6a	0.6a	0.9b	*	*	
Diethyl succinate (mg/l)	1.87a	2.04ab	2.63b	2.06ab	2.45ab	2.85b	1.86a	*		
C13-nonorisoprenoids μg/l)										

1,1,6-Trimethyl-1,2-	0.6	0.5	0.4	0.5ab	0.5b	0.3a	0.6b		**	
Vitispirane isomers	1.5	1.2	1.3	1.3	1.6	1.1	1.3			
Methoxypyrazines (ng/l)										
3-Isobutyl-2-	<2	<2	<2							
Carbonyl compounds (µg/l)										
Diacetyl	743.4	943.3	744.4	706.3	676.4	1406.7	745.5			
Furfural*1	256.1	185.8	365.8	227.3ab	501.2b	258.3ab	182.7a		*	
3,5-Dimethoxy-4-	10.1a	9.0a	18.5b	11.8	17.4	10.6	13.6	***		
5-Methylfurfural	4.4	2.7	6.0	3.0	9.5	5.5	2.3			***
5-Acetoxymethyl-2-furaldehyde	13.6a	11.7a	21.4b	16.8	16.4	15.2	13.1	***		
Syringaldehyde	89.9	78.5	116.4	86.6	118.9	94.2	103.4	**		
Lactones (µg/L)										
Delta-decalactone	4.4b	3.5a	5.0c	4.5	3.8	4.3	4.3	***		
(E)-whisky lactone	3.0b	0.8a	1.8ab	1.2	2.8	2.6	1.7	**		
(Z)-whisky lactone	2.9	2.0	2.3	1.8	4.4	2.5	1.6			
Volatile phenols (µg/L)										
4-Vinylguaiacol	122.1b	129.8b	96.4a	118.6b	114.4b	76.4a	141.9b	**	**	
2-Methoxy-4-propylphenol*1	0.5	0.3	0.3	0.2	0.3	0.4	0.6			
Eugenol	2.7	1.5	1.7	1.9	2.3	1.4	1.7			
Guaiacol	2.1b	3.4c	1.4a	2.3	2.0	2.6	2.4	***		
(E)-isoeugenol	1.7b	0.4a	1.2ab	0.8	0.8	0.7	2.3			**
m/p-Cresol isomer	2.1b	1.4a	1.0a	1.5	1.2	1.4	1.6	***		
4-methylguaiacol	0.6	0.3	0.2	0.3	0.4	0.3	0.4			
2,6-Dimethoxyphenol	3.5b	1.5a	3.2b	2.7	2.6	2.8	2.5	*		
Vanillin*1	28.4	25.1	28.5	26.1	36.7	22.4	25.4			
(Z)-isoeugneol*1	7.5b	5.4a	9.5c	7.0	8.9	8.1	7.3	***		
Ethyl vanillin	1.7b	0.8a	2.4b	1.5	1.9	2.3	1.2	***		
Acetovanillone	20.9a	24.1ab	25.7b	23.2ab	25.7ab	20.3a	27.3b		**	

Different letters in the row in the categories ´mean values vintage´ or ´mean values origin´ show significant differences in relation to a Tukey B test; * 1 The data do not reply with the requirements of the normal distribution p⩾0.05; * 2 average of five wines per vintage; * significant at level p <0.1 (F-level not shown); ** significant at level p⩽0.01 (F-level not shown); *** significant at level p⩽0.001 (F-values not shown)

Table S2. Sample list with the mean Huglin index in brackets.

Generic wineregion	Specific wineregion	samples 2015 (Huglin index)	samples 2016 (Huglin index)	samples 2017 (Huglin index)
	Neusiedlersee*1		1 (2156)	2 (2178)
Burgenland	Leithaberg	6 (2194)	6 (2156)	6 (2158)
	Südburgenland*1		1 (2156)	2 (2108)
	Weinviertel*1	6 (2145)	6 (2046)	6 (2099)
	Wagram*1	6 (2182)	4 (2070)	5 (2110)
	Wachau		1 (2051)	1 (2085)
Lower Austria	Kremstal*1		2 (2051)	2 (2100)
	Traisental*1		1 (2051)	2 (2110)
	Thermenregion	6 (2236)	6 (2136)	8 (2116)
	Kamptal*1		4 (2035)	3 (2064)
Vienna	Wien*1	6 (2202)	6 (2126)	5 (2170)
Styria	Südsteiermark	5 (2111)	6 (2001)	6 (2018)
	Vulkanland	1 (2140)	1 (2098)	2 (2070)
Sum		36 (2179)	45 (2039)	50 (2108)

Table S3: Number of typical (typicity>6), medium typical (4<typicity<6) and atypical rated wines (typicity<4) of each tasting

Wines	Panel of experts and producers			Consumer panel			trained descriptive panel		
	Typical wines	Medium typical wines	Atypical wines	Typical wines	Medium typical wines	Atypical wines	Typical wines	Medium typical wines	Atypical
vintage 2015	12	20	4						
vintage 2016	6	26	13						
Vintage 2017	19	23	10	11	25	14	28	17	6
Total wines	37	69	27						

Table S4. Information concerning calibration and validation of the volatile compound.

Volatile substance (µg/L)	Meth od	abbrevi ation	Quantif ier	Qualif ier	Internal standard	calibration area	linearity	RI	reference RI	repeatability	LOQ	LOD
(Z)-linalool oxide	FM	FM1	59	94, 111	3,4-dimethylanisole	0.9-174.3	0.998	1 070	1065-1098 a	5.8 %	0.87	0.26
(E)-linalool oxide	FM	FM2	59	94, 111	3,4-dimethylanisole	0.9-175.7	0.998	1 087	1065-1098 a	6.0 %	0.88	0.26
linalool	FM	FM3	71	93, 67	3,4-dimethylanisole	0.8-160	0.998	1 101	1074-1112 a	7.0 %	0.80	0.24
hotrienol	FM	FM4	71	82, 67	3,4-dimethylanisole	0.5-108.1	0.999	1 104	1101-1109 a	5.7 %	0.54	0.16
(Z)-rose oxide	FM	FM5	139	69, 154	3,4-dimethylanisole	0.6-113.2	0.998	1 110	1097-1121 a		0.57	0.17
(E)-rose oxide	FM	FM6	139	69, 154	3,4-dimethylanisole	0.1-10.8	0.999	1 123			0.05	0.02
(E)-limonene oxide	FM	FM7	94	67, 109	3,4-dimethylanisole	0.8-153.9	0.998	1 132			0.77	0.23
beta-terpineol	FM	FM8	93	121, 136	3,4-dimethylanisole	0.1-11.8	0.997	1 144	1127-1188 a		0.06	0.02
nerol oxide	FM	FM9	68	83	3,4-dimethylanisole	0.6-118.0	0.998	1 146	1137-1172 a	4.1 %	0.59	0.18
lavandulol	FM	FM10	69	111, 123	3,4-dimethylanisole	0.7-147.0	0.998	1 160	1140-1185 a	15.3 %	0.74	0.22
alpha-terpineol	FM	FM11	93	121, 136	3,4-dimethylanisole	0.4-86.0	0.998	1 194	1150-1224 a	13.3 %	0.40	0.12
gamma-terpineol	FM	FM12	121	136, 93	3,4-dimethylanisole	0.1-28.4	0.997	1 198	1177-1218 a		0.14	0.04
nerol	FM	FM13	69	93, 95	3,4-dimethylanisole	0.7-134.0	0.998	1 228	1204-1254 a	16.5 %	0.67	0.20
citronellol	FM	FM14	69	95, 93	3,4-dimethylanisole	0.7-148.0	0.998	1 229	1206-1238 a	12.2 %	0.74	0.22

geraniol	FM	FM15	69	93, 123	3,4-dimethylanisole	0.7-131.0	0.998	1 256	1221-1277 a	4.8 %	0.66	0.20
1-propanol (mg/L)	MV	HA1	31	42	d10-butanol	0.09-496.01	0.999	1035	1002-1073 b	8.2 %	0.010	0.003
isobutanol (mg/L)	MV	HA2	42	31	d10-butanol	0.91-91.79	0.999	1088	1043-1124 b	5.7 %	0.918	0.275
isoamyl alcohol (mg/L)	MV	HA3	70	43	d10-butanol	10.42-521.24	0.999	1206	1163-1255 b	7.8 %	10.425	3.127
1-butanol (mg/L)	MV	HA4	56	41	d10-butanol	0.06-2.79	0.998	1142	1110-1179 b	7.6 %	0.063	0.019
(Z)-3-hexen-1-ol (mg/L)	MV	C6-1	82	67, 55	d5-ethyl hexanoate	0.01-0.20	0.999	1371	1346-1426 b	11.0 %	0.010	0.003
1-hexanol (mg/L)	MV	C6-26	69	55	d13-hexanol	0.01-6.48	0.998	1352	1314-1396 b	2.8 %	0.013	0.004
butyric acid (mg/L)	MV	CA1	60	73	d7-butyric acid	0.93-18635.76	0.998	1625	1576-1670 b	7.1 %	0.932	0.280
isobutyric acid (mg/L)	MV	CA2	43	73, 88	d12-hexanoic acid	0.10-3.11	0.999	1568	1520-1608 b	10.9 %	0.104	0.031
isovaleric acid (mg/L)	MV	CA3	60	87	d13-hexanol	0.06-5.82	0.998	1657	1621-1715 b	4.8 %	0.058	0.017
hexanoic acid (mg/L)	MV	CA4	60	73, 87	d12-hexanoic acid	0.10-1.03	0.999	1845	1803-1889 b	7.0 %	0.103	0.031
octanoic acid (mg/L)	MV	CA5	115	73	d12-hexanoic acid	0.03-2.93	0.999	2055	2013-2106 b	15.0 %	0.029	0.009
decanoic acid (mg/L)	MV	CA6	129	73	d12-hexanoic acid	0.12-23.52	0.998	2281	2227-2318 b	17.9 %	0.118	0.035
diacetyl (mg/L)	MV	Carb1	43	86	d13-hexanol	0.10-19.62	0.998	1007	940-1020 b	19.3 %	0.098	0.029
ethyl acetate (mg/L)	MV	EE1	61	43	d5-ethyl octanoate	9.83-196.57	0.999	892	854-914 b	7.0 %	9.829	2.949

ethyl lactate (mg/L)	MV	EE2	45	75	d5-ethyl octanoate	0.10-51.78	0.998	1335	1316-1353 b	6.5 %	0.104	0.031
ethyl propanoate (mg/L)	MV	EE3	57	75	d5-ethyl hexanoate	9.80-980.00	0.998	952	915-976 b	10.6 %	0.010	0.003
diethyl succinate (mg/L)	MV	MiE1	101	129	d5-ethyl octanoate	0.1-9.9	0.997	1668	1658-1714 b	11.3 %	0.099	0.030
ethyl butanoate	MME	EE4	71	88, 43	d5-ethyl valerate	12.5-2500.0	0.998	802	770-818 a	3.4 %	12.50	3.75
ethyl isovalerate	MME	EE5	88	85, 57	d5-ethyl valerate	0.3-49.8	0.998	852	824-859 a	3.3 %	0.25	0.07
ethyl valerate	MME	EE6	85	88, 57	d5-ethyl valerate	0.1-10.4	0.998	901	871-929 a	4.4 %	0.13	0.04
ethyl hexanoate	MME	EE7	88	99, 43	d5-ethyl hexanoate	13.2-2641.0	0.999	1001	976-1014 a	3.1 %	13.21	3.96
ethyl heptanoate	MME	EE8	88	113	d5-ethyl valerate	0.1-10.5	0.999	1098	1080-1099 a	3.9 %	0.05	0.02
ethyl octanoate	MME	EE9	88	101, 127	d5-ethyl octanoate	16.4-3280.0	0.999	1202	1173-1202 a	2.6 %	16.40	4.92
ethyl decanoate	MME	EE10	88	101	d5-ethyl decanoate	16.9-3385.8	0.999	1399	1367-1405 a	8.2 %	16.93	5.08
ethyl dodecanoate	MME	EE11	88	101, 157	d5-ethyl decanoate	0.3-50.8	0.998	1595	1566-1596 a	15.7 %	0.25	0.08
ethyl tetradecanoate	MME	EE12	TIC	TIC	d5-ethyl decanoate	0.3-49.9	0.998	1795	1769-1799 a	11.9 %	0.25	0.07
ethyl hexadecanoate	MME	EE13	88	101, 157	d5-ethyl decanoate	3.9-55.9	0.998	1990	1966-2013 a	13.0 %	3.92	1.18
methyl isovalerate	MME	ME1	74	85, 57	d5-ethyl valerate	0.1-10.4	0.999	774	766-778 a		0.05	0.02
methyl hexanoate	MME	ME2	74	87, 43	d5-ethyl valerate	0.1-10.6	0.999	924	902-931 a	3.9 %	0.05	0.02
methyl octanoate	MME	ME3	74	87, 127	d5-ethyl hexanoate	0.1-8.7	0.999	1124	1105-1138 a	6.6 %	0.04	0.01

methyl decanoate	MME	ME4	74	87, 143	d5-ethyl octanoate	0.1-10.6	0.999	1325	1304-1329 a	5.9 %	0.05	0.02
methyl dodecanoate	MME	ME5	74	87, 143	d5-ethyl decanoate	0.2-41.9	0.998	1525	1503-1527 a		0.21	0.06
methyl tetradecanoate	MME	ME6	74	87, 143	d5-ethyl decanoate	0.2-48.6	0.998	1727	1699-1738 a		0.24	0.07
isoamyl acetate	MME	ISAE1	43	70	d5-ethyl valerate	1.0-11.6	0.996	877	851-885 a	4.1 %	0.98	0.29
isoamyl butanoate	MME	ISAE2	71	70, 43	d5-ethyl hexanoate	0.1-9.6	0.999	1057	1041-1086 a	7.2 %	0.05	0.01
isoamyl isovalerate	MME	ISAE3	85	70	d5-ethyl octanoate	0.1-9.2	0.998	1105	1081-1105 a		0.05	0.01
isoamyl hexanoate	MME	ISAE4	70	43, 99	d5-ethyl octanoate	0.1-10	0.997	1254	1238-1254 a	5.9 %	0.05	0.02
isoamyl octanoate	MME	ISAE5	70	127	d5-ethyl octanoate	0.1-10.1	0.997	1448	1450 a	13.8 %	0.05	0.02
ethyl benzoate	MME	ArE1	105	122	d5-ethyl hexanoate	0.1-14.3	0.999	1173	1138-1206 a	6.3 %	0.07	0.02
ethyl phenylacetate	MME	ArE2	91	91	d5-ethyl octanoate	0.1-13.0	0.996	1244	1209-1251 a	9.5 %	0.07	0.02
isobutyl acetate	MME	HAA1	43	56	d5-ethyl valerate	12.7-2540.0	0.998	771	741-788 a	8.3 %	12.70	3.81
2-methylbutyl acetate	MME	HAA2	43	70	d5-ethyl valerate	13.4-2680.0	0.997	878	863-892 a	3.7 %	13.40	4.02
hexyl acetate	MME	HAA3	43	56, 61	d5-ethyl hexanoate	13.2-2630.0	0.999	1013	987-1025 a	3.2 %	13.15	3.95
isobutyl propionate	MME	MiE2	57	29	d5-ethyl valerate	0.3-52.1	0.998	866	843-866 a	8.3 %	0.26	0.08
butyl isobutanoate	MME	MiE3	43	71	d5-ethyl hexanoate	1.0-11.0	0.999	953	952-955 a	9.5 %	0.98	0.30

pentyl butanoate	MME	MiE4	43	71, 70	d5-ethyl hexanoate	0.1-10.8	0.999	1095	1062-1098 a	12.1 %	0.05	0.02
hexyl 2-methylbutanoate	MME	MiE5	99	56, 117	d5-ethyl hexanoate	0.1-10.2	0.999	1237	1204-1247 a		0.05	0.02
propyl isovalerate	MME	MiE6	85	103	d5-ethyl hexanoate	0.1-10.0	0.998	950	928-951 a		0.05	0.02
butyl butanoate	MME	MiE7	71	88	d5-ethyl hexanoate	13.0-2600.0	0.999	996	969-996 a	0.9 %	13.00	3.90
propyl octanoate	MME	MiE8	145	127, 61	d5-ethyl octanoate	0.1-10.4	0.997	1292	1290 a	4.8 %	0.05	0.02
isobutyl octanoate	MME	MiE9	57	56	d5-ethyl decanoate	0.1-9.8	0.998	1349	1348-1370 a	14.9 %	0.05	0.01
vitispirane isomers	NOR	NOR1	177,1	192,1 , 149,1	d5-vitispirane isomers	0.1-31.4	0.998	1536	1507-1543 b	1.9 %	0.05	0.02
1,1,6-trimethyl-1,2-dihydronaphthalene	NOR	NOR2	157,1	172,1 , 142,1	d6-1,1,6-trimethyl-1,2-dihydronaphthalene	0.1-30.2	0.999	1723	1722-1724 b	4.9 %	0.05	0.02
3-isobutyl-2-methoxypyrazine (ng/L)	MP	MP1	124	151	d3-3-isobutyl-2-methoxypyrazine	2-50	0.999	1181	1170-1186 a	1.6 %	2.0	0.8
furfural	OV	Carb2	96	95	3,4-dimethylanisole	1.5-1500.0	0.999	835	800-848 a	5.2 %	1.50	0.45
5-methylfurfural	OV	Carb3	110	109	3,4-dimethylanisole	1.1-1100.0	0.997	965	926-987 a	5.8 %	1.10	0.33
5-acetoxymethyl-2-furaldehyde	OV	Carb4	126	109	3,4-dimethylanisole	1.3-1300.0	0.999	1309	1304 a	14.3 %	1.30	0.39
syringaldehyde	OV	Carb5	182	181	3,4-dimethylanisole	1.4-1430.0	0.999	1665	1617-1670 a	17.0 %	1.43	0.43
3,5-dimethoxy-4-hydroxyacetophen	OV	Carb6	181	196	3,4-dimethylanisole	2.0-1960.0	0.999	1739	1741-1744 a	19.0 %	1.96	0.59

(E)-whisky lactone	OV	L1	99	71	3,4-dimethylanisole	1.1-1110.0	0.998	1294	1289-1332 a	13.1 %	1.11	0.33
(Z)-whisky lactone	OV	L2	99	71	3,4-dimethylanisole	1.1-1050.0	0.999	1323	1310 a		1.05	0.32
delta-decalactone	OV	L3	99	71	3,4-dimethylanisole	1.3-1280.0	0.997	1447	1444-1447 a	10.6 %	1.28	0.38
o-cresol	OV	VP1	108	107	3,4-dimethylanisole	1.0-1040.0	0.999	1054	1029-1080 a	7.5 %	1.04	0.31
m/p-cresol isomer	OV	VP2	107	108	3,4-dimethylanisole	1.6-1550.0	0.999	1067	1105-1065 a	6.1 %	1.55	0.47
guaiacol	OV	VP3	109	124	3,4-dimethylanisole	1.1-1050.0	0.999	1090	1052-1114 a	10.1 %	1.05	0.32
4-methylguaiacol	OV	VP4	138	123	3,4-dimethylanisole	1.2-1240.0	0.998	1196	1155-1196 a		1.24	0.37
4-ethylguaiacol	OV	VP5	137	152	3,4-dimethylanisole	1.7-1650.0	0.998	1279	1243-1287 a	12.0 %	1.65	0.50
4-vinylguaiacol	OV	VP6	150	135	3,4-dimethylanisole	1.0-1020.0	0.999	1315	1272-1334 a	10.0 %	1.02	0.31
2,6-dimethoxyphenol	OV	VP7	154	139	3,4-dimethylanisole	1.6-1640.0	0.998	1349	1349-1367 a	21.6 %	1.64	0.49
eugenol	OV	VP8	164	149	3,4-dimethylanisole	1.2-1230.0	0.999	1351	1320-1397 a	5.5 %	1.23	0.37
2-methoxy-4-propylphenol	OV	VP9	137	166	3,4-dimethylanisole	1.3-1250.0	0.999	1360	1356-1382 a	22.2 %	1.25	0.38
vanillin	OV	VP10	151	152	3,4-dimethylanisole	1.5-1450.0	0.999	1393	1350-1394 a	13.5 %	1.45	0.44
(Z)-isoeugneol	OV	VP11	164	149	3,4-dimethylanisole	0.2-170.0	0.999	1401	1392-1423 a	7.9 %	0.17	0.05
(E)-isoeugenol	OV	VP12	164	149	3,4-dimethylanisole	1.1-1140.0	0.999	1453	1447-1473 a	19.2 %	1.14	0.34
ethyl vanillin	OV	VP13	137	166	3,4-dimethylanisole	2.0-1990.0	0.998	1457	1448-1453 a	18.4 %	1.99	0.60
acetovanillone	OV	VP14	151	166	3,4-dimethylanisole	2.0-1960.0	0.998	1474	1447-1480 a	5.1 %	1.96	0.59
methyl vanillate	OV	ME7	151	182	3,4-dimethylanisole	1.2-1220.0	0.998	1525	1532 a	5.9 %	1.22	0.37
ethyl vanillate	OV	EE14	151	196	3,4-dimethylanisole	1.3-1330	0.999	1577	1560-1574 a	18.4 %	1.30	0.39

(Data in empty fields could not be collected because it was not available or not detectable in the validation wine.)

a Kovats RI according to the RI calculation of Van Den Dool and Kratz, non-polar column, temperature ramp + custom temperature program (@nist database: https://webbook.nist.gov/chemistry/)
b Kovats RI according to the RI calculation of Van Den Dool and Kratz, polar column, temperature ramp + custom temperature program (@nist database: https://webbook.nist.gov/chemistry/)

FM=method for free monoterpenes, MV=method for main volatile substances, MME=method for major and minor esters, NOR=method for C13-norisoprenoids, MP=method for methoxypyrazines, OV=method for volatile phenols, oak volatiles, lactones and some carbonyl compounds

Table S5. Composition of the aroma standards for the descriptively trained panel.

**Aroma standard**	**Specification per litre of base wine**
yellow pomaceous fruit	60 ml (30 %) pear + 140 ml (70 %) apple juice
green pomaceous fruit	200 ml Granny Smith apple juice freshly pressed + 1 mg/l hexyl acetate
pear	180 ml pear juice
citrus fruits	50 ml/l lemon juice freshly pressed (organic lemons)
banana	50 ml/l banana juice +1 mg/l isoamyl acetate (acetic acid 3-methylbutyl ester)
pineapple	60 ml pineapple juice
stone fruits	100 ml apricots and peach juice each
walnut	5 drops of aroma standard
vanilla	100 mg vanillin
